# Specific brain imaging alterations underlying autistic traits in children with attention-deficit/hyperactivity disorder

**DOI:** 10.1186/s12993-023-00222-x

**Published:** 2023-11-20

**Authors:** Juan Liu, Qian-Rong Liu, Zhao-Min Wu, Qiao-Ru Chen, Jing Chen, Yuan Wang, Xiao-Lan Cao, Mei-Xia Dai, Chao Dong, Qiao Liu, Jun Zhu, Lin-Lin Zhang, Ying Li, Yu-Feng Wang, Lu Liu, Bin-Rang Yang

**Affiliations:** 1 Children’s Healthcare and Mental Health Center, Shenzhen Children’s Hospital, Shenzhen, Guangdong China; 2 Peking University Sixth Hospital/Institute of Mental Health, NHC Key Laboratory of Mental Health (Peking University), National Clinical Research Center for Mental Disorders (Peking University Sixth Hospital), Beijing, China

**Keywords:** ADHD, Autistic traits, Behavior, Resting-state functional MRI

## Abstract

**Background:**

Autistic traits (ATs) are frequently reported in children with Attention-Deficit/Hyperactivity Disorder (ADHD). This study aimed to examine ATs in children with ADHD from both behavioral and neuroimaging perspectives.

**Methods:**

We used the Autism Spectrum Screening Questionnaire (ASSQ) to assess and define subjects with and without ATs. For behavioral analyses, 67 children with ADHD and ATs (ADHD + ATs), 105 children with ADHD but without ATs (ADHD − ATs), and 44 typically developing healthy controls without ATs (HC − ATs) were recruited. We collected resting-state functional magnetic resonance imaging (rs-fMRI) data and analyzed the mean amplitude of low-frequency fluctuation (mALFF) values (an approach used to depict different spontaneous brain activities) in a sub-sample. The imaging features that were shared between ATs and ADHD symptoms or that were unique to one or the other set of symptoms were illustrated as a way to explore the “brain–behavior” relationship.

**Results:**

Compared to ADHD-ATs, the ADHD + ATs group showed more global impairment in all aspects of autistic symptoms and higher hyperactivity/impulsivity (HI). Partial-correlation analysis indicated that HI was significantly positively correlated with all aspects of ATs in ADHD. Imaging analyses indicated that mALFF values in the left middle occipital gyrus (MOG), left parietal lobe (PL)/precuneus, and left middle temporal gyrus (MTG) might be specifically related to ADHD, while those in the right MTG might be more closely associated with ATs. Furthermore, altered mALFF in the right PL/precuneus correlated with both ADHD and ATs, albeit in diverse directions.

**Conclusions:**

The co-occurrence of ATs in children with ADHD manifested as different behavioral characteristics and specific brain functional alterations. Assessing ATs in children with ADHD could help us understand the heterogeneity of ADHD, further explore its pathogenesis, and promote clinical interventions.

**Supplementary Information:**

The online version contains supplementary material available at 10.1186/s12993-023-00222-x.

## Background

Attention-Deficit/Hyperactivity Disorder (ADHD) is a common neurodevelopmental disorder that is characterized by inattention, hyperactivity, and impulsivity. The fifth edition of the *Diagnostic and Statistical Manual of Mental Disorders* (DSM-5) [1] no longer excludes comorbidity with autism spectrum disorder (ASD) from the diagnosis of ADHD. Numerous pieces of evidence suggest that ADHD and ASD might overlap in genetics, social function and interaction, executive function, and brain imaging changes [2]. Even when a diagnosis of ASD is absent, individuals with ADHD might also exhibit symptoms of autism or autistic traits (ATs) [3].

ATs represent stereotypy and deficits in social interactions, usually defined by positive results on ASD scales. Nevertheless, ATs per se do not meet the clinical threshold for ASD diagnosis [4, 5]. In young people with ADHD, the co-occurrence of ATs can significantly influence their clinical characteristics and their neuropsychological and social functions; this co-occurrence can persist into adulthood [6]. Social deficits might be more associated with oppositional symptoms, while restrictive and repetitive behaviors (RRBs) might predict symptoms of hyperactivity and impulsivity (HI) [7]. In terms of behavioral expression, ADHD probands with ATs (ADHD + ATs) have significantly more problems than those without ATs (ADHD − ATs) [6, 8]. In terms of cognitive functions, impairments in concentration, working memory, nonverbal reasoning, and visuomotor skills are more prominent in ADHD + ATs than in ADHD − ATs [7, 8]. As for social function, patients whose ADHD is comorbid with ATs demonstrate more impairments in terms of problems with spare time, activity impairments, and problems with peers [6].

In addition to the above-mentioned behavioral and cognitive evidence, some studies have tried to investigate ATs in children with ADHD based on brain imaging data to explore the potential underlying neurobiological mechanisms. Most evidence has come from studies comparing ADHD and ASD probands to explore potential overlapping and distinctive brain structural and/or functional alterations. Subcortical-volume changes might be the same between ADHD and ASD, while cortical thickness changes might or might not be the same between the two conditions [9]. Fractional anisotropy of the corpus callosum was found to play an essential role in the overlapping of ADHD and ASD symptoms [10]. ADHD and ASD both feature hypoactivation of the right anterior insula during motor response inhibition tasks [11]. Compared with healthy controls (HCs) and patients who had only ADHD, patients with comorbid ADHD and ASD exhibited decreased functional connectivity (FC) in the local right temporoparietal cortex during theory-of-mind testing [12]. The above-mentioned shared brain features of both conditions, and in particular brain changes in patients with comorbid ADHD and ASD, might help us understand ATs in children with ADHD.

Few studies thus far have directly investigated AT-related changes in brain imaging. A study of structural brain images showed a positive relationship between ASD score and gray-matter volume in adults with ADHD [13]. Although studies have identified several brain region volumes that predict ASD symptoms or affect social cognition, the strong correlations were not unique to ADHD [14, 15]. Cooper et al*.* explored white-matter (WM) microstructural characteristics of ATs in patients with ADHD. They found that AT-related changes to WM were mainly located in the right posterior limb of the internal capsule/corticospinal tract, right cerebellar peduncle, and midbrain [16], while ADHD severity was correlated with WM microstructure in the left subgenual cingulum [17]. Zhang et al*.* found that cerebral blood flow in the left middle frontal gyrus was correlated with both ASD and ADHD scores, while that in the left temporal pole was negatively correlated with ASD score, in children and adolescents with comorbid ADHD and ATs. However, their post hoc analysis revealed nonsignificant differences in these clusters between the ADHD groups with and without ATs [18]. One study showed that higher social impairment and more ADHD symptoms in adolescents with ADHD correlated with functional dysconnectivity among the default mode network (DMN), frontoparietal network, and cingulo-opercular network [19]. The above-mentioned studies notwithstanding, evidence from the existing literature is still insufficient to illustrate AT-specific brain changes in ADHD, which might be independent from those correlated with core ADHD symptoms.

Resting-state functional magnetic resonance imaging (rs-fMRI) is a non-invasive tool used to probe the spontaneous neural activity of intrinsic human-brain functional organizations, which likely reflects disorder etiology [20]. Amplitude of low-frequency (0.01–0.08 Hz) fluctuation (ALFF), an index for measuring changes in resting-state blood oxygen level–dependent signals, can relatively and indirectly reflect spontaneous brain activity [21, 22]. It has been widely used in studies on mental disorders [21–25], with its high test–retest reliability in gray matter regions and high sensitivity for discerning differences between individuals and groups [26]. ALFF has successfully identified cognitive, executive-dysfunction, and methylphenidate treatment–related brain changes in ADHD [27–29]. Taken together, this evidence suggests that ALFF is a sensitive method for detecting spontaneous brain activity in ADHD.

In summary, ATs are common in ADHD and can affect the behavioral, cognitive, and brain features of the disorder. To our knowledge, the neuroimaging mechanism of ATs has not been well illustrated. Therefore, in the present study we aimed to investigate ATs in children with ADHD in terms of both behavioral expression and brain functional alterations to explore the “brain–behavior” relationship, which could promote better understanding of AT-related heterogeneity in children with ADHD. To analyze brain functional alterations, the ALFF in rs-fMRI was used. We hypothesized that ATs in children with ADHD would be associated with more ADHD symptoms and with specific brain imaging changes.

## Results

Demographic and clinical characteristics of the two groups (HC − ATs, ADHD) or three groups (HC − ATs, ADHD − ATs, and ADHD + ATs) are listed in Table 1. No significant differences were found in gender distribution no matter between the two groups or among the three groups. Children in the HC − ATs group were older than ADHD overall group (9.51 ± 1.16 *vs.* 8.84 ± 1.71; *P* = 0.0027) and ADHD − ATs (9.51 ± 1.16 *vs.* 8.89 ± 1.70; *P* = 0.0353), ADHD + ATs (9.51 ± 1.16 *vs.* 8.75 ± 1.75;* P* = 0.0164) subgroups. Full-Scale Intelligence Quotient (FSIQ) scores were higher in HC − ATs than ADHD overall group (106.34 ± 9.52 *vs.* 93.33 ± 10.36; *P* = 1.200E^−12^) and in ADHD − ATs (106.34 ± 9.52 *vs.* 94.24 ± 9.43; *P* = 2.754E^−10^), ADHD + ATs (106.34 ± 9.52 *vs.* 91.90 ± 11.60; *P* = 4.979E^−12^) subgroups. No difference was found between ADHD + ATs and ADHD − ATs in distributions of age, FSIQ, or ADHD subtypes.

**Table 1:** Comparisons of demographic characteristics, ASSQ and SNAP-IV scores of the different groups

	ADHD (N = 172)	HC − ATs^1^ (N = 44)	*t/F/χ*^2 a^	*P*^a^	ADHD − ATs^2^ (N = 105)	ADHD + ATs^3^ (N = 67)	*F/χ*^2 b^	*P*^b^	Pairwise comparison ^c^
Age in years (mean ± SD)	8.84 ± 1.71	9.51 ± 1.16	3.07	0.0027	8.89 ± 1.70	8.75 ± 1.75	3.18	0.0436	1 > 2 = 3
Male [n, (%)]	146 (84.89)	33 (75.00)	2.41	0.1205	88 (83.81)	58 (86.57)	2.63	0.2684	–
FSIQ (mean ± SD)	93.33 ± 10.36	106.34 ± 9.52	7.56	1.200E^−12^	94.24 ± 9.43	91.90 ± 11.60	29.78	3.929E^−12^	1 > 2 = 3
ADHD subtypes [n, (%)]									
ADHD-IA	72 (41.86)	–	–	–	50 (47.62)	22 (32.84)	–	0.1209	–
ADHD-HI	7 (4.07)	–	–	–	3 (2.86)	4 (5.97)			
ADHD-C	93 (54.07)	–	–	–	52 (49.52)	41 (61.19)			
ASSQ (mean ± SD)									
Total score	10.41 ± 8.11	3.03 ± 2.74	10.50	0.0014	5.09 ± 3.67	18.76 ± 5.78	235.16	2.499E^−54^	1 = 2 < 3
Social interaction	3.99 ± 3.33	0.95 ± 1.06	13.21	0.0004	1.97 ± 1.77	7.16 ± 2.67	152.00	1.522E^−41^	1 = 2 < 3
Communication problems	2.76 ± 2.54	1.23 ± 1.60	1.61	0.2066	1.37 ± 1.39	4.94 ± 2.39	81.74	5.549E^−27^	1 = 2 < 3
RRBs	3.66 ± 3.28	0.84 ± 1.01	10.46	0.0014	1.74 ± 1.75	6.66 ± 2.83	129.36	2.433E^−37^	1 = 2 < 3
SNAP-IV (mean ± SD)									
Total score	30.40 ± 9.63	17.98 ± 10.85	39.05	2.247E^−9^	29.14 ± 10.09	32.36 ± 8.55	21.88	2.338E^−9^	1 < 2 < 3
Inattention	16.95 ± 4.90	10.02 ± 5.84	44.02	2.690E^−10^	16.45 ± 5.09	17.75 ± 4.51	23.35	6.958E^−10^	1 < 2 = 3
HI	13.44 ± 5.69	7.95 ± 5.25	25.28	1.000E^−6^	12.70 ± 6.11	14.61 ± 4.78	15.30	6.260E^−7^	1 < 2 < 3

*SD* standard deviation. *FSIQ* Full-Scale Intelligence Quotient. *ADHD-IA* ADHD with inattention; *ADHD-HI* ADHD with hyperactivity and impulsivity; *ADHD-C* a combination of both. *ASSQ* Autism Spectrum Screening Questionnaire. *RRBs* restrictive and repetitive behaviors. *SNAP-IV* Swanson, Nolan, and Pelham rating scale, version IV. HI: hyperactivity and impulsivity

^a^With two sample *t* test, *χ*^2^ test, or general linear model analysis after controlling for FSIQ, age, and gender, between HC − ATs and ADHD

^**b**^With analysis of variance (ANOVA), *χ*^2^, or analysis of covariance (ANCOVA) after controlling for FSIQ, age, and gender, among HC − ATs, ADHD − ATs and ADHD + ATs groups, with Fisher’s exact test between ADHD − ATs and ADHD + ATs groups

^c^with least-significant-difference (LSD) *t*-test among the three groups

### Behavioral analyses

For AT symptoms, totals and sub-scores of the Autism Spectrum Screening Questionnaire (ASSQ), were significantly higher in children with ADHD than in HC − ATs, except for the communication problems (Table 1). Interestingly, when we defined subgroups for ADHD based on their ATs (ADHD − ATs and ADHD + ATs), we found higher ASSQ totals and all sub-scores in ADHD + ATs than in other two groups including the communication problem; No significant difference was found between ADHD − ATs and HC − ATs.

For ADHD core symptoms, totals and sub-scores of the Swanson, Nolan, and Pelham rating scale, version IV (SNAP-IV) were significantly higher in children with ADHD than in HC − ATs. Post hoc analyses among the three groups showed that SNAP-IV totals and sub-scores were higher in the two ADHD groups than in HC − ATs. In our comparison between subjects with ADHD, ADHD + ATs had more HI symptoms than ADHD − ATs (Table 1), whereas no difference was found in symptoms of inattention.

Based on the above findings, we further explored the relationship between HI symptoms and ATs in children with ADHD using partial-correlation analysis after controlling for age, sex, and FSIQ. HI symptoms showed significant positive correlation with social-interaction score (*r* = 0.299, *P* = 7.7E^−05^), communication problems (*r* = 0.196, *P* = 0.0105), and RRBs (*r* = 0.192, *P* = 0.0124). However, the above relationship was not seen in HC − ATs (all *P* > 0.05).

To address the potential influence of sex on our present findings, we used two sample *t* test between males and females in children with ADHD. The results indicated no difference between males (n = 146) and females (n = 26) in AT symptoms, including the ASSQ totals (10.39 ± 7.90 *vs.* 10.54 ± 9.38; *t* = − 0.09, *P* = 0.9320) and all the three subscales: social interaction (3.99 ± 3.33 *vs.* 4.00 ± 3.42;* t* = − 0.01, *P* = 0.9923), communication problem (2.75 ± 2.48 *vs.* 2.81 ± 2.86; *t* = − 0.10, *P* = 0.9203), RRBs (3.64 ± 3.23 *vs.* 3.73 ± 3.61; *t* = − 0.12, *P* = 0.9012). In addition, no sex difference was found for ADHD core symptoms, including the SNAP-IV totals (30.69 ± 9.70 *vs.* 28.73 ± 9.23; *t* = 0.96, *P* = 0.3400) and the two subscales: inattention (17.00 ± 4.99 *vs.* 16.65 ± 4.48; *t* = 0.34, *P* = 0.7362), HI (13.69 ± 5.73 *vs.* 12.08 ± 5.36; *t* = 1.33, *P* = 0.1853). Further, we also found no differences in totals and all sub-scores of ASSQ and SNAP-IV scales between males and females in both ADHD − ATs and ADHD + ATs subgroups (all *P* > 0.05).

### Imaging analyses

Demographic and clinical characteristics of subjects included in the imaging analyses are shown in Additional file 1: Table S1.

### Categorical comparisons

**Fig. 1:**
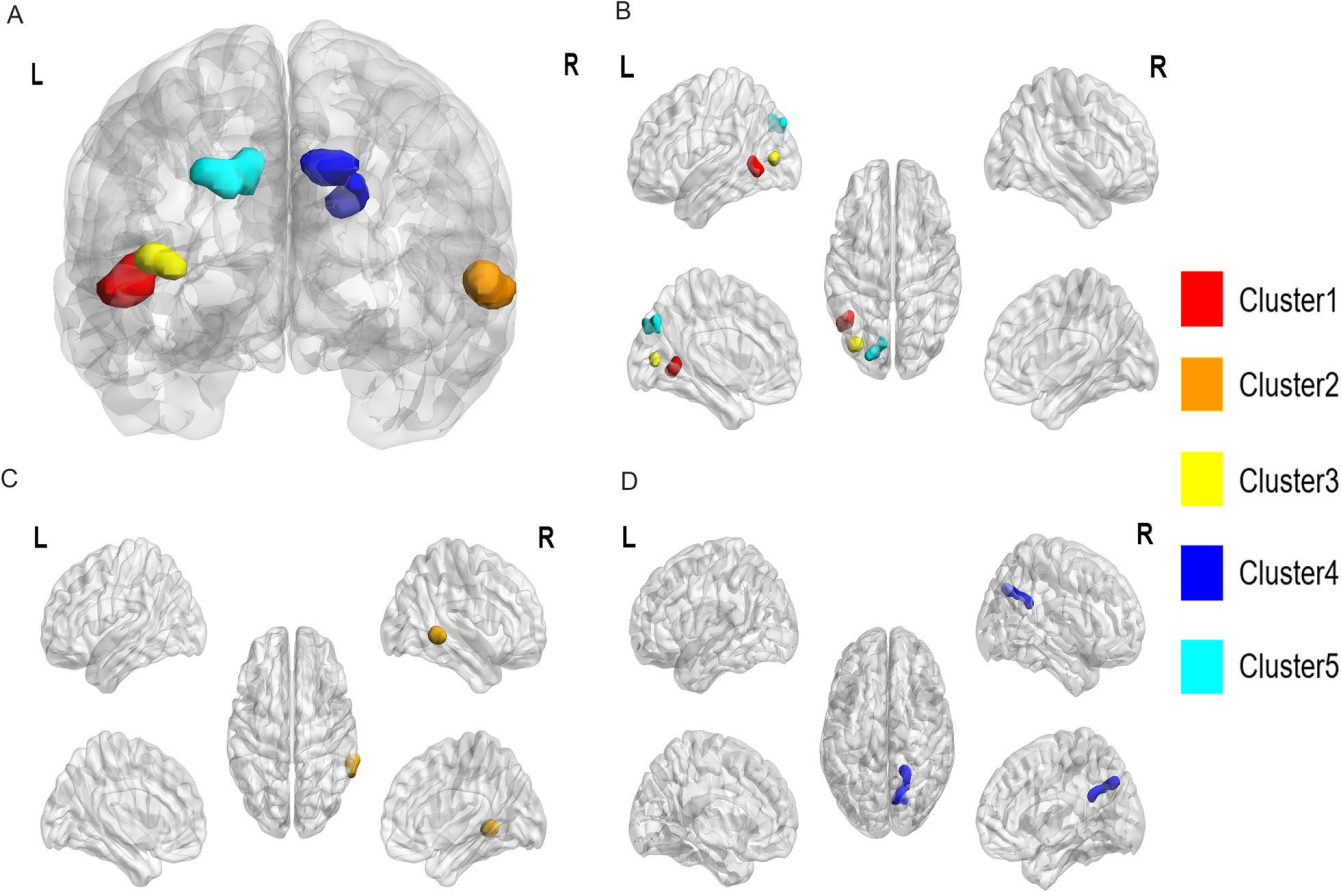
Differences in mALFF values among the three groups (three-dimensional [3D] view; N = 102). **A** Significant brain regions in the three groups. **B** Brain regions specifically related to ADHD. **C** Brain regions specifically related to ATs. **D** Brain regions involved in both ADHD and ATs, with analysis of covariance (ANCOVA) and post hoc group comparisons of mALFF values after controlling for FSIQ, age, and gender; or establishing multiple-regression equation by multiple linear-regression analyses between mALFF clusters and behaviors in ADHD. Cluster 1 (red): left MTG; Cluster 2 (orange): right MTG; Cluster 3 (yellow): left MOG; Cluster 4 (dark blue): right PL/precuneus; Cluster 5 (light blue): left PL/precuneus. *mALFF* mean amplitude of low-frequency fluctuations. *MTG* left middle temporal gyrus. *MOG* middle occipital gyrus. *PL* parietal lobe

Five clusters indicated group differences in mean ALFF (mALFF) values: in the left middle temporal gyrus (MTG; *F* = 10.259,* P* = 9.200E^**−**5^), right MTG (*F* = 8.249,* P* = 4.950E^**−**4^), left middle occipital gyrus (MOG; *F* = 7.787,* P* = 7.350E^**−**4^), right parietal lobe (PL)/precuneus (*F* = 8.939, *P* = 2.750E^**−**4^), and left PL/precuneus (*F* = 7.428, *P* = 0.0010; Table 2, Fig. 1A). Post hoc analyses indicated potential shared and distinctive brain functional alterations between ADHD and ATs.

**Table 2:** Comparison of mALFF values among the three groups (N = 102)

mALFF	Brain regions	Peak coordinates (mm)			*F* ^a^	*P* ^a^	Pairwise comparisons					
Clusters		X	Y	Z			ADHD + ATs *vs.* HC − ATs		ADHD − ATs *vs.* HC − ATs		ADHD + ATs *vs.* ADHD − ATs	
							LSD *t*-value	*P1*	LSD *t*-value	*P2*	LSD *t*-value	*P3*
Cluster 1	Left MTG	− 48	− 57	3	10.259	9.200E^−5^	3.677	3.990E^−4^	4.130	6.800E^−5^	0.271	0.7853
Cluster 2	Right MTG	60	− 42	0	8.249	4.950E^−4^	3.829	2.250E^−4^	0.839	0.4105	3.478	7.910E^−4^
Cluster 3	Left MOG	− 33	− 78	6	7.787	7.350E^−4^	2.679	0.0084	3.884	2.060E^−4^	− 0.521	0.6008
Cluster 4	Right PL/precuneus	12	− 69	36	8.939	2.750E^−4^	0.796	0.4241	3.972	1.380E^−4^	− 2.700	0.0082
Cluster 5	Left PL/precuneus	− 9	− 75	39	7.428	0.0010	2.758	0.0069	3.718	3.280E^−4^	− 0.326	0.7434

*mALFF* mean amplitude of low-frequency fluctuations. *MTG* middle temporal gyrus. *MOG* middle occipital gyrus. *PL* parietal lobe

^a^With analysis of covariance (ANCOVA) after controlling for Full-Scale Intelligence Quotient (FSIQ), age, and gender;* P*: Overall significance of ANCOVA in the three groups; *P1–3*: Significance of post hoc test with least-significant-difference (LSD) *t*-test

*ADHD-specific alterations:* Higher mALFF values in the left MTG (Cluster 1), left MOG (Cluster 3), and left PL/precuneus (Cluster 5) might be specifically associated with ADHD (Figs. 1B and Additional file 1: Fig. S1). These were the only differences we identified between the ADHD groups and HC − ATs, whereas we saw no differences between ADHD + ATs and ADHD − ATs (Table 2).

*AT-specific alterations*: Higher mALFF values in the right MTG (Cluster 2) might be unique to ATs. These were the only differences we found between ADHD + ATs and the other two groups, but a nonsignificant difference was found between ADHD − ATs and HC − ATs (Fig. 1C and Additional file 1: Fig. S1, Table 2).

*Alterations shared between ADHD and ATs:* Changes in mALFF values in the right PL/precuneus (Cluster 4) might be common to both ADHD and ATs, albeit in diverse associated directions (Figs. 1D and Additional file 1: Fig. S1). As shown in Table 2, Cluster 4 mALFF values in ADHD − ATs were higher than those in HC − ATs and ADHD + ATs, whereas no difference was found between ADHD + ATs and HC − ATs.

### Quantitative relationship between brain functional alterations and clinical symptoms

We used multiple linear-regression analyses to further explore the quantitative relationship among ASSQ, SNAP-IV scores, and the mALFF values of the five different brain regions in categorical (group comparison) analysis of the ADHD groups. SNAP-IV and ASSQ total scores were set as covariates when analyzing the association of ATs and ADHD core symptoms with brain functional alterations, respectively.

We found ATs to be negatively correlated with mALFF values in the right PL/precuneus (Cluster 4) on all three subscale scores (*P* = 0.0006, 0.0073, and 0.0004 for social interaction, communication problems, and RRBs, respectively). In contrast, mALFF values in the right MTG (Cluster 2) were found to correlate positively with RRBs (*P* = 0.0035) and communication problems (marginally, *P* = 0.0620; Table 3). We did not find ADHD-specific brain regions that had any effects on ADHD symptoms.

**Table 3:** Independent variables of mALFF clusters related to SNAP-IV and ASSQ sub-scores in children with ADHD (N = 45)

Dependent variable	Independent variable	Standardized coefficient	*t*-value	*P1*	*r*^2^/*r*^2^adj	*P*
Inattention	Intercept	–	6.09	< 0.0001	0.1524/0.1120	0.0311
	Left MTG	− 0.2116	− 1.48	0.1456		
	Sex	− 0.3083	− 2.16	0.0365		
HI	Intercept	–	− 0.87	0.3907	0.1160/0.0954	0.0221
	FSIQ	0.3406	2.38	0.0221		
Social interaction	Intercept	–	5.37	< 0.0001	0.2438/0.2262	0.0006
	Right PL/precuneus	− 0.4937	− 3.72	0.0006^***^		
Communication problems	Intercept	–	2.86	0.0066	0.3769/0.3146	0.0007
	Right MTG	0.2415	1.92	0.0620		
	Right PL/precuneus	− 0.3758	− 2.83	0.0073^**^		
	FSIQ	− 0.2109	− 1.57	0.1251		
	Sex	− 0.3057	− 2.41	0.0209		
RRBs	Intercept	–	2.14	0.0379	0.4210/0.3786	< 0.0001
	Right MTG	0.3707	3.10	0.0035^**^		
	Right PL/precuneus	− 0.4616	− 3.87	0.0004^***^		
	Sex	− 0.2360	− 1.98	0.0544		

*mALFF* mean amplitude of low-frequency fluctuations. *SNAP-IV* Swanson, Nolan, and Pelham rating scale, version IV. *ASSQ* Autism Spectrum Screening Questionnaire. *HI* hyperactivity and impulsivity. *RRBs* restrictive and repetitive behaviors. *FSIQ* Full-Scale Intelligence Quotient. *MTG* middle temporal gyrus. *PL* parietal lobe. *r*^2^: determination coefficient; *r*^2^adj: adjusted determination coefficient, *P*: overall significance of multiple stepwise linear regression, *P1:* significance of standardized partial regression coefficient. ^**^P < 0.01, ^***^P < 0.001

## Discussion

Our present study attempted to explore ATs in children with ADHD from both behavioral and brain imaging perspectives. The results indicated that children with ADHD + ATs presented more global impairment in all aspects of autistic symptoms and had more HI symptoms. The brain functional changes measured by mALFF in the left MTG, left MOG, and left PL/precuneus might be specifically related to ADHD. Furthermore, those in the right MTG might be closely related to ATs, and those in the right PL/precuneus were correlated with both ADHD symptoms and ATs, albeit in diverse directions. Quantitative analyses supported the relationship of altered mALFF in the right MTG and right PL/precuneus with ATs in children with ADHD.

Approximately 20–65% of children with ADHD exhibit symptoms of ASD [8, 30]. In this study, the prevalence of ATs as assessed via ASSQ was nearly 39%. Interestingly, scores of communication problems were not different between ADHD and HC − ATs; however, when we separated ATs from ADHD and compared ASSQ totals and sub-scores among HC − ATs, ADHD − ATs, and ADHD + ATs, we found higher scores of communication problems in ADHD + ATs than in other two groups. It elucidated the importance of evaluating ATs in children with ADHD, which can help us better identify the subgroup of ATs who may not meet the criteria for ASD diagnosis and draw attention to future targeted intervention on ATs in ADHD. As indicated in the post hoc group comparisons, the ADHD-AT group did not differ from HC − ATs in any of the three subdomains. This suggested ASSQ had good sensitivity in identifying ADHD + ATs and supported its use for future clinical screening of ATs in children with ADHD. The ADHD + AT subgroup demonstrated impairments in all three subdomains, which was consistent with previous studies [31, 32]. In terms of correlation between ATs and ADHD core symptoms, we found HI symptoms to be more severe in ADHD + ATs than in ADHD − ATs, whereas no difference was found for inattention, as discussed in Grzadzinski’s study [33]. This could not be explained by a different subtype diagnosis in ADHD between the two ADHD groups, as this subtype diagnosis was not different in our present study. In addition, HI symptoms were found to be positively correlated with all ASSQ subdomains in children with ADHD but not in HC − ATs. One study showed that ASD traits could be predicted by more HI symptoms, but not by symptoms of inattention, in ADHD [34], supporting the potential relationship between HI symptoms and ATs. In the existing literature, HI symptoms are commonly reported to be more closely correlated with RRBs, while inattention is associated with all subdomains of autistic symptoms [32, 35, 36]. Further investigation using network analysis might be worth performing to identify key symptoms that act as “bridges” linking HI and ATs [37], which could help explain the inconsistency of research results between HI symptoms and the subdomains of ATs to some extent.

In our imaging analyses, the first interesting finding was the specific association between functional changes in the right MTG and ATs in both categorical and quantitative analyses. The mALFF values of AT-specific changes in the right MTG showed significant positive association with RRBs and a positive trend association with communication problems. Much evidence has supported the involvement of the right MTG in ASD/ATs. Task fMRI studies have proven that activation of the right MTG is positively correlated with autism symptom severity in people with ASD or ATs [38, 39]. Disrupted FCs within the MTG or between the MTG and other regions of the brain were detected in subjects with ASD [40–42]. No similar study has reported on the quantitative relationship between mALFF values of the right MTG and RRBs. Greater ALFF values in the right MTG have been shown to be related to greater visuomotor variability in ASD [43]. It would be interesting to further explore whether functional alterations in the right MTG would influence RRBs via visuomotor-related uncoordinated limb movement.

Although the left MTG’s abnormal activity has also been reported in ASD [44–46] and correlated with ASD symptoms in subjects with ADHD comorbid with ATs [18], our present findings suggested that changes in mALFF values of the left MTG should be ADHD specific. The MTG is an important part of the visual-attention network [47], the dysfunction of which plays an important role in ADHD etiology [48]. Much evidence from brain functional and structural analyses has emphasized the role of the left MTG in ADHD [49–52]. Other studies have drawn inconsistent conclusions on whether activation of the bilateral MTG is related to ADHD [53–55]. Interestingly, after separating ATs from ADHD, we found that right lateralization of the MTG was related to ATs and left lateralization thereof was related to ADHD. Whether right asymmetry of ATs in ADHD affects left–right brain asymmetry in ADHD might be another discussion point in the future.

Another AT-related brain functional change was found in the right PL/precuneus, which was supported by both categorical and quantitative analyses. The precuneus is part of the superior parietal lobules and a key node of the posterior DMN, and it plays a crucial role in social functions such as the self-reference cognitive process and theory-of-mind [56]. Decreased FC between the right precuneus and other brain regions has been found in ASD [40, 57]. In addition to rs-fMRI evidence, studies on brain structure [58–60] and using task-fMRI [61, 62] emphasize the role of the right precuneus in ASD. Despite the heterogeneity of the DMN in different studies, findings generally indicate hypoactivation of DMN nodes in ASD [63]. More interestingly, the direction of mALFF in the right PL/precuneus was reverse associated with ADHD and ATs, as shown in the categorical comparison. The combination of these two opposite directions resulted in a nonsignificant difference between ADHD + ATs and HC − ATs. The normalization of functional alteration in the right PL/precuneus in subjects with ADHD and ATs prompted us to wonder whether ATs in children with ADHD would yield some benefit in clinical expression, cognitive function, or other social functions. It is worth conducting further studies to explore this important issue. We should note that several studies have found precuneus abnormalities to be involved in both ADHD and ASD, but in the same direction of effect [64, 65]. Therefore, we will enroll typically developing children with ATs in our future research to confirm the accuracy of the effect direction of the shared right-PL/precuneus cluster in ADHD and ATs. In the left PL/precuneus, mALFF alterations were ADHD specific, which was consistent with a previous report [66]. Together with the right caudate nucleus, the left precuneus can indicate ADHD in normal groups with 62.52% accuracy [67]. Interventional experiments showed that larger left-precuneus volumes correlated with poor methylphenidate response [68].

In addition to the above-mentioned brain regions, we found a relatively specific correlation between brain functional alteration in the left MOG and ADHD. The MOG is the part of the occipital lobes that contains most of the visual cortex and has been demonstrated to be involved in modulation of unconscious processes by category-selective attention [69]. A recent study showed that Granger causality analysis values from the ventral putamen to the left MOG were significantly negatively correlated with HI symptoms in patients with ADHD [70]. Rs-fMRI and diffusion tensor imaging studies also support a relationship between changes to the left MOG and ADHD [53, 54].

In summary, our behavioral data demonstrated the existence of ATs in children with ADHD, illustrating the structure of autistic symptoms and their strong correlation with HI symptoms. ATs are not temporary but remain stable for many years in ADHD [6]. Identifying ATs in children with ADHD would be helpful for targeted interventions. For example, in children with ADHD co-occurring with ATs, behavioral intervention into HI might help improve their social-interaction performance. ATs in children with ADHD might be marked by specific brain functional changes, which would help us understand neuroimaging heterogeneity in these children. Autism is thought to be a natural form of human diversity, due to the continuity from ATs in the general population to ASD in patients. The AT-specific brain cluster changes we found might also help us understand the characteristics of brain functional alterations in individuals with comorbid ADHD and ASD according to DSM-5 criteria. In addition, the shared and distinct features we identified for ADHD and ATs might offer some potential interventional targets.

There were several limitations in the current study. First, it focused on ATs in children with ADHD; we did not recruit HC children with ATs. The recruitment of typically developing subjects with ATs would help yield a more granular description of specific brain alterations indicative of ATs in the general population. Second, we used only mALFF in rs-fMRI as the brain functional feature for analyses. Future studies should acquire multimodal data to deeply explore potential shared and distinctive brain functional alterations between ADHD and ATs. Third, children with ASD only, those with ADHD only, and comorbid groups should be recruited to explore continuous detailed changes in behavior, cognitive function, and brain alterations in these populations, from ATs to ASD spectrum in ADHD. Fourth, our study was not matching the age and IQ between HC and ADHD groups, though we put them as covariates in our statistically analysis to control the two confounding factors. Finally, our small sample size with its relatively narrow age range might not have covered the whole population or all development periods of ADHD; we also found no correlations between ADHD symptoms and mALFF in the left MTG, left MOG, or left PL/precuneus. Larger samples including multiple age ranges should be enrolled to address this issue.

## Conclusions

Among children with ADHD, some subjects also exhibit autistic traits. In this study, subjects with ADHD + ATs showed different behavioral characteristics and potentially specific brain functional alterations. Assessment and exploration of ATs in children with ADHD could help us understand the heterogeneity of ADHD, better explore its pathogenesis, and promote clinical intervention.

## Methods

### Participants and procedures

For children with ADHD, all subjects were recruited in the clinics of Shenzhen Children’s Hospital (SCH; Shenzhen, China). For HCs, they were recruited from different local elementary schools in Shenzhen by advertisements. A total of 172 medication-naïve patients with clinically diagnosed ADHD based on the DSM-IV from the outpatient department of SCH from June 2017 to December 2020, and 45 HCs were recruited. This cross-sectional study was approved by the Medical Ethics Committee of SCH, and informed consent was obtained from all participants and their parents.

Patients came to the clinic on their first visit. They underwent a semi-structured clinical interview that used the Kiddie Schedule for Affective Disorders and Schizophrenia Present and Lifetime Version (K-SADS-PL) to confirm the diagnosis of ADHD and exclude other psychiatric disorders [71]. Inclusion criteria for children with ADHD were as follows: (1) meeting the diagnosis of ADHD; (2) age 6–16 years; (3) FSIQ assessed using the Wechsler Intelligence Scale for Children, fourth edition ≥ 70; and (4) without any drug, behavioral, or psychological intervention for ADHD. Exclusion criteria were as follows: (1) ASD or sleep disorder diagnosis; (2) tic disorders, intellectual disability, conduct disorder, schizophrenia, affective disorder, or other psychiatric disorder; and (3) physical illnesses and neurological disorders such as epilepsy, short stature, congenital heart disease, enuresis, or immune encephalitis. HCs who were willing to participate our project came to our clinic for inclusion criteria and the exclusion criteria evaluation based on the clinical interview using K-SADS-PL. Any present or lifetime diagnosis of any psychiatric disorder led to exclusion. Other inclusion and exclusion criteria for HCs were the same as those for the ADHD group.

All participants’ parents were asked to complete the SNAP-IV and ASSQ scales for ADHD and ASD symptom evaluation. Subjects with a total score of ASSQ ≥ 12 were defined as having ATs [72]. The ADHD and HC groups was further divided into two subgroups separately, ADHD + ATs, ADHD–ATs and HC + ATs, HC–ATs. Ultimately, only one HC combined with ATs, that cannot be one group, so we excluded this single one for comorbidity with ATs and kept the left three groups (ADHD + ATs, ADHD − ATs and HC − ATs) for analysis.

For the imaging analyses, only subjects who agree to participate and met the following criteria were included for data acquisition: (1) right-handed; (2) do not have metal implants (including non-removable dentures); (3) not suffering from claustrophobia. Any visible abnormalities on MRI images as examined by an experienced radiologist (*e.g.*, cysts) during MRI scans, or excessive head motion with > 3 mm of translation or > 3° of rotation in any direction, led to exclusion for brain analysis. Five children with ADHD were excluded for further imaging analyses due to their excessive head motion.

Finally, 67 ADHD + ATs, 105 ADHD − ATs, and 44 HC − ATs were included for behavioral analyses; and 21 ADHD + ATs, 38 ADHD − ATs, and 43 HC − ATs for imaging analyses.

## Measurements

### Swanson, Nolan, and Pelham rating scale, version IV

We used the SNAP-IV parent form to evaluate ADHD symptoms. The form includes 18 items on a three-point rating scale (0 = “not at all,” 1 = “just a little,” 2 = “quite a bit,” and 3 = “very much”). The Chinese version of SNAP-IV has been demonstrated to be a reliable and valid instrument, with satisfactory reliability (all Cronbach’s *α* coefficients of SNAP-IV sub-scales > 0.88) and sufficient sensitivity (0.87) and specificity (0.79) [73].

### Autism spectrum screening questionnaire

The ASSQ consists of 27 items on a three-point rating scale (0 = “normal,” 1 = “some abnormality,” and 2 = “definite abnormality”). Nine items were designed for social interaction, 7 for communication problems, and 11 for RRBs [72]. The ASSQ has been proven to have good internal consistency (Cronbach’s *α* = 0.86) [74]. To define the ADHD + AT group in our study, we used the Mandarin Chinese version, whose cutoff value of 12 can distinguish individuals with ASD from unaffected controls [72].

## Resting-state functional MRI

### Data acquisition

We acquired rs-fMRI data of children at SCH using a Siemens Skyra scanner (Siemens Healthcare, Forchheim, Germany) with a standard 12-channel head coil. Functional images were acquired using an echo-planar imaging sequence with the following parameters: repetition time = 2000 ms, echo time = 30 ms, flip angle = 90°, thickness/skip = 3.5/0.7 mm, matrix = 64 × 64, field of view = 200 × 200 mm, 33 axial slices, 240 volumes, and 3 × 3 mm in-plane resolution. All participants were asked to close their eyes without falling asleep during the 8 min of fMRI scanning.

### Data processing

We acquired resting-state functional images using RESTplus [75] on the MATLAB R2014a platform. Data preprocessing included the following steps: excluding the first 10 time points, slice timing, realignment, segmentation of each participant’s images to the echo planar imaging standard template and then normalization to the Montreal Neurological Institute space (resampled voxel size = 3 × 3 × 3 mm^3^), spatial smoothing with a 6-mm full-width-at-half-maximum Gaussian filter, linear detrending, and nuisance covariate regression (including the friston 24-parameter model, WM signal, and cerebrospinal-fluid signal) [76].

For higher test–retest reliability in gray matter regions and more sensitive for discerning differences between individuals and groups [26], ALFF, rather than fALFF, was chosen to identify our hypothesis. After preprocessing, we calculated ALFF. Mean ALFF (mALFF; ALFF at each voxel normalized by the mean ALFF of voxels in gray matter) was used for statistical analyses.

## Statistical analysis

For behavioral data, analysis of two sample *t*-test, or variance (ANOVA) and post hoc analysis with the least-significant-difference (LSD) *t*-test, *χ*^2^ test, or Fisher’s exact test were used to compare the demographic and clinical characteristics of the HC − ATs and ADHD two groups or HC − ATs, ADHD − ATs and ADHD + ATs three groups. We used general linear-model analysis with sex, age, and FSIQ as covariates, or analysis of covariance (ANCOVA) and post hoc analysis with the LSD *t*-test, to compare AT symptoms and ADHD symptoms between subjects with ADHD and HC − ATs or among the three groups. Partial-correlation analysis with sex, age, and FSIQ as covariates was used to separately explore the relationships between AT symptoms and ADHD symptoms in the ADHD and HC − ATs groups.

For neuroimaging data, we used ANCOVA and RESTplus to compare the mALFFs of children in the ADHD + AT, ADHD − AT, and HC − AT groups, with sex, age, and FSIQ as covariates. The multiple-comparison correction was based on Analysis of Functional NeuroImages’s AlphaSim, with a threshold of* P* < 0.01 and cluster size ≥ 42 [77]. We then extracted mALFF signals from each significant cluster to perform the following post hoc analysis using the LSD *t*-test: relationships between mALFF values of different brain regions and symptoms as assessed by ASSQ and SNAP-IV were investigated using stepwise multiple linear-regression analyses, after elimination of factors whose variance inflation factor values were > 10.

We performed all behavior and mALFF value statistical analyses using SAS version 9.1 (SAS Institute, Cary, NC, USA) and SPSS version 23 (IBM Corp., Armonk, NY, USA). *P* < 0.05 was considered significant.

### Supplementary Information

**Additional file 1:** **Table S1.** Demographic characteristics of groups for neuroimaging analysis. **Figure S1.** Differences in mALFF values among the three groups (two-dimensional [2D] view; N = 102).

## Data Availability

The datasets presented in this article are not readily available due to regulations governing ethical approval.

## Abbreviations

ADHD: Attention-deficit/hyperactivity disorder
ATs: Autistic traits
ADHD + ATs: Children with ADHD and ATs
ADHD − ATs: Children with ADHD but without ATs
ASD: Autism spectrum disorder
ASSQ: Autism Spectrum Screening Questionnaire
ANOVA: Analysis of variance
ANCOVA: Analysis of covariance
ALFF: Amplitude of low-frequency fluctuation
DMN: Default mode network
DSM: *Diagnostic and Statistical* *Manual* *of Mental Disorders*
FC: Functional connectivity
FSIQ: Full-Scale Intelligence Quotient
HI: Hyperactivity/impulsivity
HCs: Healthy controls
HC − ATs: Healthy controls but without ATs
K-SADS-PL: Kiddie-Schedule for Affective Disorders and Schizophrenia Present and Lifetime Version
MOG: Middle occipital gyrus
MTG: Middle temporal gyrus
MRI: Magnetic resonance imaging
mALFF: Mean ALFF
PL: Parietal lobe
rs-fMRI: Resting-state functional MRI
RRBs: Restricted and repetitive behaviors
SNAP-IV: Swanson, Nolan, and Pelham rating scale, version IV
WM: White matter
