# Supplementary material for: Specific brain imaging alterations underlying autistic traits in children with attention-deficit/hyperactivity disorder
Source: Behav Brain Funct. 2023 Nov 20;19:20. doi: 10.1186/s12993-023-00222-x (PMC10658985; doi:10.1186/s12993-023-00222-x)
Supplement: Supplementary file 1 — Additional file 1: Table S1. Demographic characteristics of groups for neuroimaging analysis. Figure S1. Differences in mALFF values among the three groups (two-dimensional [2D] view; N = 102). [file 12993_2023_222_MOESM1_ESM.docx]

### Additional file

## Table S1 Demographic characteristics of groups for neuroimaging analysis

|  | **HC−ATs^1^**  **(N = 43)** | **ADHD−ATs^2^**  **(N = 38)** | **ADHD+ATs^3^**  **(N = 21)** | ***F/χ*^2 a^** | ***P*** | **Pairwise comparison ^b^** |
| --- | --- | --- | --- | --- | --- | --- |
| **Age in years (mean ± SD)** | 9.63 ± 1.25 | 9.56 ± 1.13 | 9.32 ± 1.42 | 0.44 | 0.6479 | — |
| **Male [n, (%)]** | 31 (72.09) | 30 (78.95) | 20 (95.24) | 4.63 | 0.0987 | — |
| **FSIQ (mean ± SD)** | 105.88 ± 9.40 | 96.39 ± 8.68 | 95.10 ± 8.90 | 15.22 | 2.000E^−6^ | 1 > 2 = 3 |
| **ADHD subtypes [n, (%)]** |  |  |  |  |  |  |
| ADHD-IA | — | 24 (63.16) | 11 (52.38) | — | 0.5806 | — |
| ADHD-HI | — | 0 (0.00) | 0 (0.00) |  |  |  |
| ADHD-C | — | 14 (36.84) | 10 (47.62) |  |  |  |
| **Framewise displacement (mm)** | 0.49 ± 0.20 | 0.54 ± 0.28 | 0.42 ± 0.16 | 1.79 | 0.1727 | — |

SD: standard deviation. FSIQ: Full-Scale Intelligence Quotient. ADHD-IA: ADHD with inattention, ADHD-HI: ADHD with hyperactivity and impulsivity, ADHD-C: a combination of both. **^a^** With analysis of variance (ANOVA) or *χ*^2^/Fisher’s exact test; **^b^** with least-significant-difference (LSD) *t-*test.


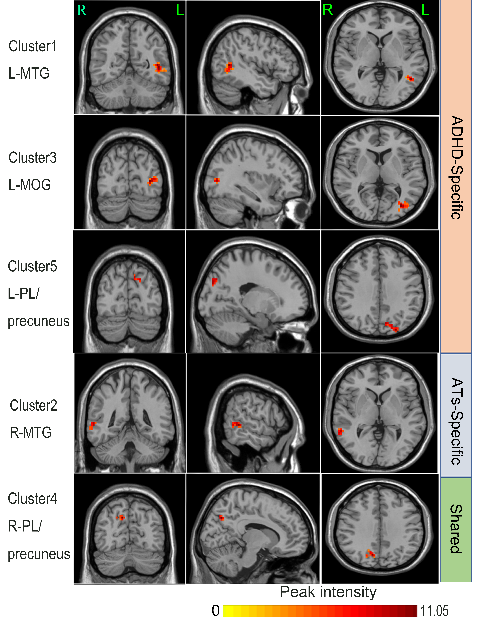


**Figure S1** Differences in mALFF values among the three groups (two-dimensional [2D] view; N = 102). The mALFF values of the L-MTG, L-MOG, and L-PL/precuneus were specifically related to ADHD. The mALFF values of the R-MTG were specifically related to ATs. The mALFF values of the R-PL/precuneus were involved in both ADHD and ATs. With analysis of covariance (ANCOVA) and *post hoc* group comparisons, or multiple linear-regression analyses between mALFF clusters and behaviors in ADHD. mALFF: mean amplitude of low-frequency fluctuations. L-MTG: left middle temporal gyrus. L-MOG: left middle occipital gyrus. L-PL/precuneus: left parietal lobe/precuneus. R-MTG: right middle temporal gyrus. R-PL/precuneus: right parietal lobe/precuneus.
